# Calcitonin gene‐related peptide (CGRP) in epilepsy: Mechanistic insights from preclinical models

**DOI:** 10.1002/epi4.70363

**Published:** 2026-11-13

**Authors:** Marina Romozzi, Luca Pani, Cristina Benatti, Catello Vollono, Jacopo Canonichesi, Serenella Servidei, Paolo Calabresi, Fabio Tascedda, Luigi Francesco Iannone

**Affiliations:** ^1^ Dipartimento Universitario di Neuroscienze Università Cattolica del Sacro Cuore Rome Italy; ^2^ Dipartimento di Neuroscienze, Organi di Senso e Torace Fondazione Policlinico Universitario Agostino Gemelli IRCCS Rome Italy; ^3^ Department of Biomedical, Metabolic, and Neural Science University of Modena and Reggio Emilia Modena Italy; ^4^ Department of Psychiatry and Behavioral Sciences University of Miami Miami Florida USA; ^5^ Università Telematica San Raffaele di Roma Rome Italy; ^6^ San Raffaele Sulmona Sulmona L'Aquila Italy; ^7^ Department of Life Sciences University of Modena and Reggio Emilia Modena Italy

**Keywords:** CGRP, cortical hyperexcitability, epilepsy, migraine comorbidity, neuroinflammation

## Abstract

Calcitonin gene‐related peptide (CGRP) is a pleiotropic neuropeptide widely expressed in the central and peripheral nervous systems, with established roles in neurovascular regulation, synaptic transmission, and neuroinflammation. While CGRP has been extensively characterized in migraine pathophysiology, its role in epilepsy remains incompletely defined and is primarily supported by experimental evidence. Preclinical studies consistently demonstrate CGRP upregulation following prolonged seizures, particularly in models of status epilepticus, and suggest that CGRP signaling may contribute to key processes implicated in epileptogenesis. These include modulation of neuronal excitability, blood–brain barrier permeability, and neuroimmune responses, such as microglial activation. At the same time, CGRP has been shown to engage neuroprotective pathways, including anti‐apoptotic and anti‐oxidative mechanisms, as well as barrier‐stabilizing effects, supporting a complex and context‐dependent role. Shared pathophysiological features between migraine and epilepsy, such as cortical hyperexcitability, neuroinflammation, and cortical spreading depolarization, provide a conceptual framework in which CGRP may act as a convergent mechanistic mediator. However, direct evidence in human epilepsy remains limited, and the translational relevance of these findings requires careful interpretation. This review provides a focused synthesis of mechanistic insights from experimental models, highlighting the dual role of CGRP in seizure‐related processes and delineating current gaps in knowledge. Potential clinical implications, including the effects of CGRP‐targeting therapies, are discussed in a hypothesis‐generating context, with emphasis on the need for rigorous studies to clarify their relevance in epilepsy.

**Plain Language Summary:**

Migraine and epilepsy sometimes occur together and share changes in brain activity. This review examines CGRP, a signaling molecule involved in migraine, and its possible role in seizures. Animal studies suggest that CGRP can either increase abnormal brain activity and inflammation or protect nerve cells, depending on the conditions. Early observations in people raise the possibility that migraine medicines blocking CGRP could also affect seizures, but they do not prove a benefit. Further research is needed to determine whether these medicines can safely reduce seizures or prevent epilepsy.

**Key points**

- Preclinical studies suggest CGRP can either promote neuronal hyperexcitability or protect neurons, depending on context.
- Most evidence comes from acute experimental models, limiting conclusions about long‐term epilepsy development.
- Preliminary observational findings suggest possible seizure benefits of CGRP blockade but cannot establish efficacy.
- Limited brain penetration complicates the translation of CGRP‐targeted migraine therapies to epilepsy.
- Prospective studies are needed to establish seizure‐related efficacy and safety of CGRP‐targeting therapies.

## 1 INTRODUCTION

Calcitonin gene‐related peptide (CGRP) is a 37‐amino‐acid neuropeptide with potent vasodilatory and neuro‐modulatory properties, widely expressed in the central (CNS) and peripheral nervous systems (PNS). It acts mainly through a heteromeric receptor complex composed of the calcitonin‐like receptor (CLR), receptor activity‐modifying protein 1 (RAMP1), and receptor component protein (RCP).^1^, ^2^ CGRP signaling regulates vascular tone, neurogenic inflammation, and synaptic activity, linking peripheral trigeminovascular activation to central excitability. Its role in migraine has been extensively characterized, leading to the development of therapeutic antagonists for migraine.^1^, ^3^

Emerging evidence suggests that CGRP may also influence epileptogenesis. Experimental data from rodent models demonstrate that CGRP is markedly upregulated in the brain following sustained seizures or excitotoxic insults, such as kainic acid‐induced status epilepticus (SE), suggesting a link between peptide release and seizure‐related neuroinflammation.^4^, ^5^ Direct cortical application of CGRP in rodents induces epileptiform discharges, blood–brain barrier (BBB) disruption, and microglial activation, indicating that excessive CGRP signaling can create a hyperexcitable and inflamed cortical environment, which may contribute to seizure generation.^6^

Conversely, other studies highlight neuroprotective and reparative properties of CGRP, including anti‐oxidative, anti‐apoptotic, and barrier‐stabilizing effects following neuronal stress, which can be particularly relevant in epileptogenesis and possibly in epilepsy.^7^, ^8^ This dualistic profile suggests that CGRP may contribute to neuronal hyperexcitability during the acute ictal phase, while also being involved in the activation of compensatory mechanisms that promote neuroprotection and network homeostasis.^7^, ^8^ Emerging evidence also suggests that the effects of CGRP on neuronal excitability may depend on its anatomical distribution and signaling context.

Recently, migraine treatment has been reshaped by monoclonal antibodies (mAbs) and small‐molecules (gepants) targeting the CGRP pathway. These therapies are now widely used and supported by robust evidence of effectiveness and a generally favorable safety profile across diverse patient populations, including individuals with other neurological comorbidities.^9^, ^10^, ^11^, ^12^, ^13^ Still, evidence regarding the use of anti‐CGRP therapies in patients with migraine and comorbid epilepsy remains limited. This is exemplified by the SAFE‐CGRP study, which evaluated individuals with migraine treated with anti‐CGRP mAbs and presenting with complex comorbidities.^14^ In that cohort, only 4 of 487 comorbidity “cases” (0.82%) had epilepsy, and no specific epilepsy‐related outcomes were reported.^14^ Interestingly, preliminary observational data suggest that individuals with comorbid epilepsy and migraine treated with CGRP inhibitors experience fewer episodes of SE.^15^

This review provides a focused synthesis of mechanistic insights from experimental models, highlighting the dual role of CGRP in seizure‐related processes and delineating current gaps in knowledge. Potential clinical implications, including the effects of CGRP‐targeting therapies, are discussed.

## 2 MIGRAINE AND EPILEPSY: A BIDIRECTIONAL RELATION

Migraine and epilepsy are among the most prevalent and disabling disorders, both characterized by recurrent, transient, and paroxysmal neurological disturbances.^10^, ^16^ These conditions are closely related, sharing genetic susceptibility, overlapping symptomatology, and partially convergent therapeutic approaches.^17^ Epidemiological data consistently demonstrate a bidirectional association, whereby individuals affected by one disorder exhibit an increased risk of developing the other.^17^ Primary headache disorders, in particular migraine, are highly prevalent among people with epilepsy (PwE). Additional studies report that up to 79% of PwE experience recurrent headache disorders, with migraine occurring in approximately 20%–25% and tension‐type headache (TTH) in up to 40% of cases.^18^ This frequent comorbidity appears to reflect overlapping pathophysiological mechanisms, including cortical hyperexcitability, ion channel dysfunction, altered neurotransmission, and neuroinflammatory processes.^10^, ^11^, ^12^, ^13^

Migraine aura, which affects approximately one‐third of individuals with migraine, and focal epileptic seizures can present with overlapping transient neurological manifestations, particularly visual, sensory, and language symptoms, complicating the differential diagnosis. Migraine aura typically has a gradual onset with progressive spread over several minutes, whereas seizures are usually sudden in onset, brief, and highly stereotyped.^17^

The overlap between migraine and epilepsy is also formally acknowledged in the third edition of the International Classification of Headache Disorders (ICHD‐3), which includes specific diagnostic categories for seizure‐related headache, namely migraine aura‐triggered seizure, ictal epileptic headache, and post‐ictal headache.^19^, ^20^ Among these entities, post‐ictal headache represents the most prevalent syndrome in PwE.^20^ A more debated entity is migraine aura‐triggered seizure, historically referred to as “*migralepsy*.” According to the ICHD‐3, this condition is defined as an epileptic seizure occurring during or within 1 h after a migraine aura in a patient with a history of migraine with aura. This definition is based on the hypothesized pathophysiological overlap between migraine and epilepsy, particularly the role of cortical spreading depolarization (CSD) in promoting cortical hyperexcitability and facilitating seizure occurrence (*see paragraph below*).

Lastly, the close relationship between migraine and epilepsy is also evident at the therapeutic level. Several antiseizure medications (ASMs), including topiramate and valproic acid, are widely used as migraine preventive treatments.^21^, ^22^ However, their use is limited by relevant safety concerns, particularly in women of childbearing age, as well as by drug–drug interactions and systemic adverse effects.^23^ Moreover, other migraine preventive therapies may be suboptimal in PwE: calcium channel blockers and beta‐blockers can exacerbate somnolence or psychiatric comorbidities, while tricyclic antidepressants and other antidepressants are associated with a potential, although low, risk of lowering the seizure threshold.^22^, ^24^

## 3 SHARED PATHOPHYSIOLOGICAL MECHANISMS BETWEEN MIGRAINE AND EPILEPSY: CSD, TRIGEMINAL SYSTEM AND SEIZURES

Migraine aura is a reversible neurological phenomenon that manifests with visual, sensory, speech, and motor focal neurologic symptoms, usually lasting 5–60 min, either preceding or accompanying headache pain.^20^ CSD, originally described by Leão in 1944 in a rabbit model, is a wave of depolarization of neuronal and glial cells spreading across the cerebral cortex at a rate of 2–5 mm/min, followed by a long period of hyperpolarization, and has been proposed as the pathophysiological mechanism of migraine aura.^25^

The depolarization typical of CSD is associated with local ionic shifts and neurotransmitter and metabolite release.^26^ This includes massive increases in extracellular potassium and increases in intracellular sodium and calcium, and the release of glutamate.^26^ Among the neurotransmitters, glutamate plays a prominent role in initiating and propagating CSD by activating *N*‐methyl‐d‐aspartic acid (NMDA) receptors.^27^, ^28^ Vascular reactivity and blood flow modifications follow the change in neuronal excitability, with an increase in regional cerebral blood flow for 3–5 min, followed by hypoperfusion (spreading oligemia) lasting for approximately 1–2 h.^29^, ^30^

Experimental evidence shows that CSD activates trigeminovascular neurons with a temporal profile resembling the onset of headache after aura.^31^ This suggests that activation of the trigeminovascular system (TGVS) is crucial for initiating migraine attacks.^31^ The activation leads to the release of different neuropeptides, such as CGRP. The resultant arteriolar vasodilation, neurogenic inflammation, and mast cell degranulation may contribute to the activation or sensitization of dural nociceptors.^32^, ^33^, ^34^ However, it is still debated whether a relationship between CSD mechanisms and subsequent head pain exists.^34^

Evidence consistent with CSD was later demonstrated in humans using functional magnetic resonance imaging (fMRI)^35^, ^36^ and recently in a case report, the first known example of CSD captured via direct intracranial electroencephalography (EEG).^37^ In this case, a 32‐year‐old woman with a history of migraine underwent a presurgical evaluation for comorbid epilepsy. During implantation for stereotactic electroencephalography, with 94 electrode contacts distributed across both hemispheres, she experienced a typical migraine with aura. Stereotactic EEG revealed low‐voltage suppression originating in the left mesial occipital cortex and propagating anteriorly at approximately 3 mm/min, corresponding clinically to a contralateral right superior scintillating scotoma and ipsilateral headache. Intracranial stereotactic electroencephalographic recordings captured CSD during migraine with aura.^37^ Replication in other patients is necessary to establish generalizability.

Seizures represent fast, intermittent synchronization of neural networks with highly variable EEG activation patterns.^38^ Thus, CSD and epileptic seizures are distinct neurophysiological phenomena characterized by different electrical dynamics and propagation patterns. However, increasing experimental and clinical evidence indicates that they may interact in a context‐dependent manner. The thresholds for CSD and seizures are not fixed or independent and may overlap in hyperexcitable or structurally compromised brain tissue, as comprehensively reviewed by Aiba and Noebels.^38^

Several temporal relationships between seizures and CSD have been described. Electrocorticographic analyses showed heterogeneous patterns, including CSD immediately following prolonged seizures, alternating sequences of repeated CSDs and ictal activity, seizures triggering recurrent CSDs at adjacent electrodes, and, in some cases, interruption of ongoing seizures coinciding with the occurrence of CSD.^38^, ^39^, ^40^ Similar observations in conditions of brain injury further support the notion that seizures and CSD can co‐occur and interact rather than represent mutually exclusive events.^40^

Experimental studies have demonstrated that CSD can facilitate epileptiform activity under conditions of reduced inhibitory tone or pre‐existing network hyperexcitability.^41^ Using combined amygdala‐hippocampus‐cortex slices from adult Wistar rats, the authors applied a low concentration of the GABAA receptor antagonist bicuculline that alone did not induce epileptiform field potentials (EFP). SD was then induced by focal KCl application while simultaneous intra‐ and extracellular recordings were obtained from neocortical layer V neurons, hippocampal CA1 pyramidal cells, and lateral amygdala projection neurons. In control conditions, SD did not trigger EFP, but under partial GABAA disinhibition, SD consistently elicited epileptiform bursts that culminated into ictal activity within 10–30 min across cortical and subcortical regions. SD produced a large transient depolarization followed by sustained changes in intrinsic membrane properties, including depolarized resting membrane potential, decreased action potential duration, reduced after‐hyperpolarization amplitude and duration, decreased input resistance, and increased firing frequency. These results may indicate that CSD can increase neuronal excitability and promote synchronization in predisposed tissue.^41^

At the same time, evidence from controlled experimental models suggests the opposite, with CSD transiently suppressing ongoing ictal activity. Using focal neocortical seizures induced by topical 4‐aminopyridine, penicillin G, or bicuculline in mice, combined with electrocorticography and intrinsic optical imaging, the study by Tamim et al.^42^ shows that seizures frequently culminate in SD, particularly when seizure power in the δ band and spatial generalization are high. Once triggered, SD immediately terminates ictal activity and suppresses seizure activity for at least 30 min, beyond the transient electrophysiological depression classically associated with SD. Exogenously induced SD, via KCl or optogenetics, reproduced this robust and lasting antiseizure effect, whereas pharmacological suppression of SD with MK‐801 (a potent, selective, noncompetitive NMDA receptor channel blocker) enhanced seizure generalization and intensity. Similar seizure‐terminating SDs were observed in a familial hemiplegic migraine type 1 knock‐in model and during systemic kainate‐induced generalized seizures. These data may support a bidirectional interaction in which intense, spatially expanding seizure activity triggers SD, and SD in turn limits seizure propagation.^42^

Overall, current evidence supports the view that CSD and seizures represent distinct but dynamically interacting manifestations of cortical network dysfunction. Their relationship appears bidirectional and strongly dependent on the underlying pathological substrate, rather than reflecting a uniform protective or pro‐convulsant role of CSD across conditions.^38^ Within this framework, the concept of migraine aura‐induced seizures can be reinterpreted in light of the shared pathophysiology between CSD and epileptic activity.^43^ Migraine aura is widely accepted to reflect CSD propagating across susceptible cortical regions, particularly originating in the occipital cortex. In predisposed individuals, the transient hyperexcitability at the leading edge of the CSD wave, characterized by elevated extracellular glutamate, potassium accumulation, and altered inhibitory–excitatory balance, may facilitate the transition from CSD to epileptiform discharges.^41^, ^43^

Finally, regarding the connection between TGVS and seizures, a preclinical study in rats^44^ investigated whether epileptic seizures can directly or indirectly activate the TGVS and specific fibers involved in migraine pathophysiology. Seizures were induced by topical cortical application of picrotoxin (a noncompetitive antagonist of the GABAA receptor). Single‐unit recordings were obtained from peripheral meningeal nociceptors in the trigeminal ganglion (Aδ and C fibers) and from second‐order neurons in the spinal trigeminal nucleus (wide dynamic range, WDR, and high‐threshold, HT neurons). Neuronal firing was recorded for 1 h before and 2 h after seizure induction. Seizure reliably activated a substantial proportion of trigeminovascular neurons, with activation occurring in 54% of Aδ fibers, 50% of C fibers, 68% of WDR neurons, and 39% of HT neurons. In activated neurons, firing rates increased approximately twofold to fourfold compared with baseline. Some neurons showed early activation compatible with ictal headache, whereas others displayed delayed and prolonged activation compatible with postictal headache.^44^ Critically, topical application of lidocaine to the dura prevented activation of both peripheral and central trigeminovascular neurons without affecting seizure induction or maintenance. These findings suggest that all classes of peripheral and central trigeminovascular neurons are involved in the initiation and maintenance of seizure induced headache, through the activation of meningeal nociceptors, rather than solely on descending cortical mechanisms.^44^

## 4 CGRP IN EPILEPTOGENESIS AND NEURONAL EXCITABILITY: MECHANISTIC INSIGHTS

CGRP is a neuropeptide widely distributed in the nervous system, with receptors (the CGRP receptor complex) present on many neurons, non‐neural cells (such as glial and Schwann cells), and vascular structures in the brain^2^, ^45^ and strictly interacting with different pathways and receptors.^2^, ^46^ CGRP has multiple, sometimes paradoxical, roles in the brain's response to stress and injury. Experimental studies show that CGRP can induce pro‐excitatory and pro‐inflammatory effects by promoting pathological hyperexcitability in cortical networks (Table 1 summarizes the main preclinical models on CGRP and seizure/epilepsy‐related mechanisms). For example, acute application of CGRP to the rodent cortex triggered epileptiform discharges, indicating increased neuronal excitability.^4^, ^6^ In the same experimental conditions, 4 h after application, CGRP induced neuroinflammatory alterations, leading to plasma extravasation (BBB leakage) and microglial activation in the cortex, as indicated by a reduction in their ramified processes.^6^ Therefore, elevated CGRP can drive a pathophysiological state of neuronal hyperexcitability and neuroinflammation in the brain.

**TABLE 1:** Summary of preclinical studies on CGRP and seizure/epilepsy‐related mechanisms.

References	Study	Experimental model	Experiment type/intervention	Main result
^4^	Park et al. (2013)	C57BL/6 α‐CGRP+/+ and α‐CGRP−/− mice; outbred ICR mice	i.c.v. KA (0.03–0.07 μg); α‐CGRP+/+ vs. α‐CGRP−/− comparison; α‐CGRP mRNA assessment; sumatriptan pretreatment	KA ↑ hippocampal α‐CGRP mRNA; KA‐induced CA3 neuronal death was reduced in α‐CGRP−/− mice; sumatriptan (250–500 ng) attenuated neuronal death induced by KA.
^5^	Saria et al. (1989)	Male Sprague–Dawley rats	i.p. KA (10 mg/kg); i.p. PTZ (35 mg/kg); i.v. mannitol (1.5 g/kg)	↑ CGRP‐like immunoreactivity after KA, peaking at 3 days (frontal cortex +1290%, striatum +900%, dorsal hippocampus +400%, amygdala/pyriform cortex +135%); no change after PTZ; KA‐related CGRP increase prevented by mannitol.
^6^	Gimeno‐Ferrer et al. (2022)	Male Wistar rats; male C57BL/6N mouse brain slices	Topical cortical CGRP ± olcegepant; KCl‐evoked CSD; CGRP application on ex vivo brain slices	CGRP ↑ epileptiform discharges, plasma extravasation and microglial changes; effects prevented by olcegepant.
^8^	Li et al. (2026)	C57BL/6 mice; HT22/PC12 cells	Intrahippocampal KA; i.p. CGRP (50 μg/kg); glutamate‐induced neuronal injury in vitro	↓ seizure frequency/severity, neuroinflammation and neuronal damage; ↑ cognitive function; ↓ P2RX7 via inhibition of JAK1–STAT1 pathway.
^47^	Wang et al. (2022)	Male Sprague–Dawley rats	Lithium‐pilocarpine + NTG; AAV‐BDNF overexpression in vlPAG	↓ spontaneous seizure frequency/duration and ↓ CGRP immunoreactivity in TNC and TG after BDNF overexpression.
^48^	Tringali et al. (2018)	Male Wistar rat brainstem explants (in vitro)	Perampanel (0.01–100 μM); KCl‐, veratridine‐, and capsaicin‐stimulated CGRP release	↓ basal CGRP release; ↓ KCl‐ and veratridine‐stimulated CGRP release; no effect on capsaicin‐stimulated CGRP release.

Abbreviations: AAV, adeno‐associated virus; BDNF, brain‐derived neurotrophic factor; CGRP, calcitonin gene‐related peptide; CSD, cortical spreading depolarization; i.c.v., intracerebroventricular; i.p., intraperitoneal; i.v., intravenous; JAK1, Janus kinase 1; KA, kainic acid; KCl, potassium chloride; NTG, nitroglycerin; P2RX7, P2X purinoceptor 7; PTZ, pentylenetetrazol; STAT1, signal transducer and activator of transcription 1; TG, trigeminal ganglion; TNC, trigeminal nucleus caudalis; vlPAG, ventrolateral periaqueductal gray.

In line with this, high levels of CGRP have been observed after seizures. In a preclinical study,^5^ rats that experienced intraperitoneal injection of 10 mg/kg kainic acid–induced SE showed a massive increase in CGRP levels (4–13‐fold above baseline) in multiple brain regions (i.e., cortex, hippocampus, striatum) a few days after the seizures. Notably, this CGRP increase occurred after prolonged, injurious seizures but not after brief generalized seizures (no CGRP change was seen 3 days after a short pentylenetetrazol [PTZ] seizure).^5^ This suggests that CGRP upregulation is part of the brain's response to intense or sustained seizure activity, possibly in conjunction with seizure‐induced tissue injury.

In C57BL/6 mice, the intracerebroventricular (i.c.v.) administration of 0.07 μg kainic acid induced excitotoxic injury primarily affecting CA3 pyramidal neurons and significantly increasing hippocampal α‐CGRP mRNA levels, with a peak at 6–12 h and return to baseline by 24 h. Histological analysis showed marked CA3 pyramidal cell loss after kainic acid administration.^4^ This neuronal death was significantly reduced in α‐CGRP knockout mice compared to wild‐type controls. Additionally, i.c.v. pretreatment with higher doses of sumatriptan (250–500 ng) significantly attenuated kainic acid–induced neuronal loss, whereas low‐dose sumatriptan (100 ng) had no effect.^4^

CGRP release might thus contribute to epileptogenesis, as recurrent seizures and neuroinflammation form a vicious cycle. Elevated CGRP could further excite neural circuits and promote inflammatory cascades, facilitating the development of seizures. Conversely, it might also reflect an intrinsic protective response (*see below*), highlighting the complex role of CGRP in epilepsy (Figure 1).

**FIGURE 1:**
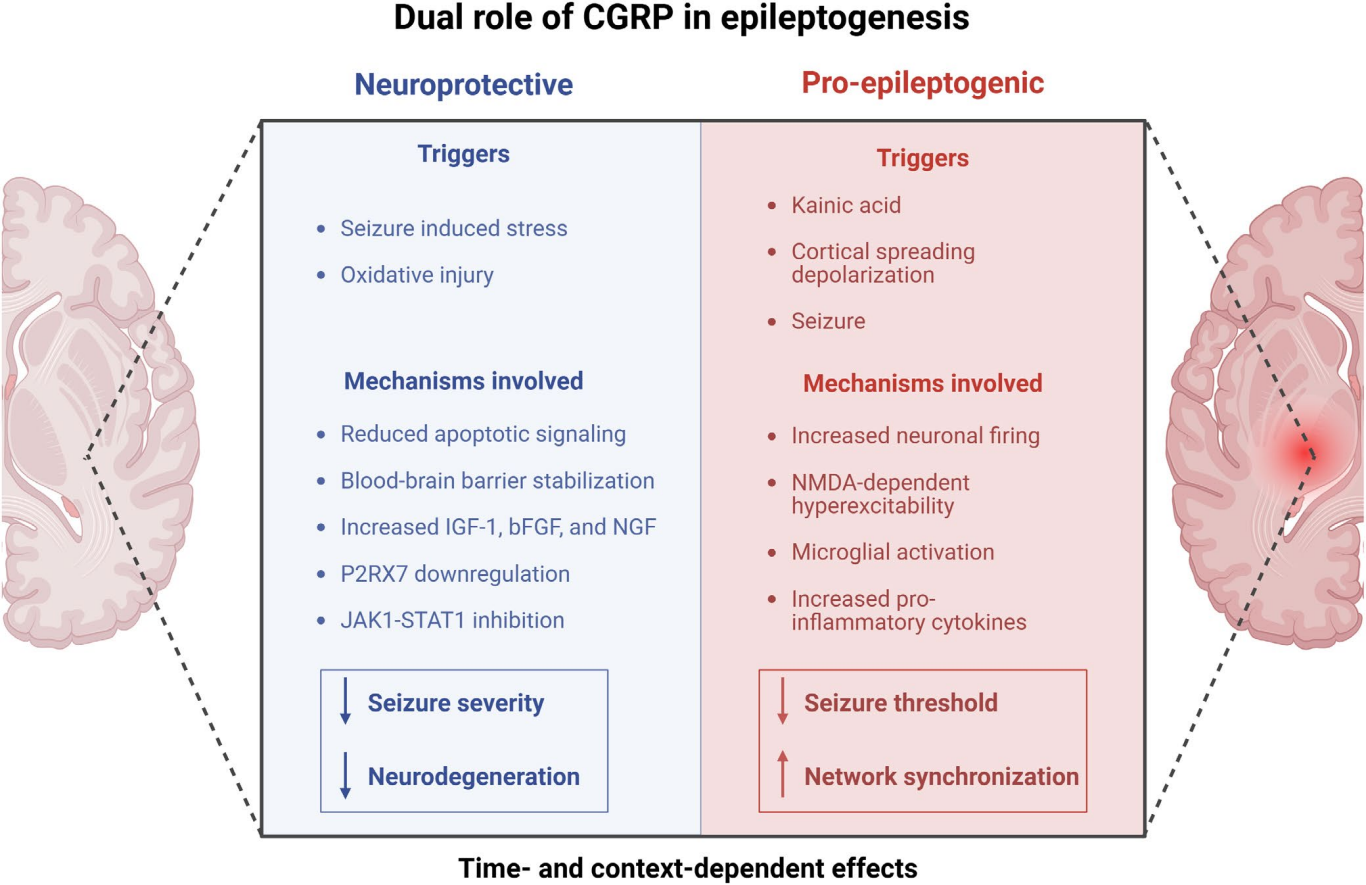
Dual and context‐dependent role of CGRP in epileptogenesis: neuroprotective versus pro‐epileptogenic mechanisms. CGRP signaling is time‐, dose‐, and context‐dependent, exerting either detrimental or protective effects depending on the underlying pathological state, timing, dose, and spatial distribution within the central nervous system. bFGF, basic fibroblast growth factor; CGRP, calcitonin gene‐related peptide; IGF‐1, insulin‐like growth factor 1; JAK1, Janus kinase 1; NGF, nerve growth factor; NMDA, *N*‐methyl‐d‐aspartate; P2RX7, purinergic receptor P2X7; STAT1, signal transducer and activator of transcription 1.

Indeed, CGRP also activates a host of neuroprotective and reparative processes following neurological stress, and it is up‐regulated by factors such as ischemia, trauma, hyperthermia, and seizures, and in turn triggers multiple neuroprotective pathways, as reported in a recent review.^7^ In fact, CGRP signaling in the brain regulates calcium influx to prevent excitotoxicity, activates anti‐apoptotic pathways, and promotes neurotrophic factors such as insulin‐like growth factor‐1 (IGF‐1), basic fibroblast growth factor (bFGF), and nerve growth factor (NGF).^7^ Interestingly, an early study using a systemic kainate model of limbic seizures induced by intraperitoneal administration (10 mg/kg), a sustained increase was observed of bFGF mRNA in the vulnerable CA1 hippocampal region up to 14 days after severe seizure activity, suggesting that bFGF induction may represent a protective or adaptive response to excitotoxic neuronal injury.^49^

Furthermore, another preclinical study,^47^ investigated whether overexpression of brain‐derived neurotrophic factor (BDNF) in the ventrolateral periaqueductal gray (vlPAG) modulates behavioral and neuropathological features in a rat model of epilepsy–migraine comorbidity. The authors used an adeno‐associated virus (AAV) vector to deliver BDNF into the vlPAG prior to inducing epilepsy with lithium–pilocarpine (127 mg/kg i.p., then 24 h later and 340 mg/kg i.p. to induce SE, respectively) and migraine‐like attacks with repeated nitroglycerin administration. BDNF overexpression significantly reduced spontaneous seizure frequency and duration, increased pain thresholds, and attenuated migraine‐like behaviors compared with control groups. Histologically, AAV–BDNF treatment reduced neuronal damage and apoptosis in the vlPAG (HE and TUNEL staining). Immunofluorescence confirmed increased BDNF and TrkB expression in the vlPAG following viral delivery. Additionally, BDNF overexpression decreased c‐Fos–positive neurons in the trigeminal nucleus caudalis and reduced CGRP immunoreactivity in both the trigeminal nucleus caudalis and trigeminal ganglion, indicating attenuation of TGVS activation. The findings suggest that vlPAG dysfunction contributes to epilepsy–migraine comorbidity and that activation of the BDNF–TrkB pathway in this region exerts both antiepileptic and analgesic effects.^47^

CGRP also reduces cerebral edema and oxidative stress after injury. Outside the BBB, CGRP also has protective vascular and immune functions.^2^ It can stabilize the endothelium, strengthen the BBB, and limit leukocyte infiltration into the CNS.^2^ These actions could mitigate some long‐term damage from seizures (e.g., limiting post‐seizure inflammation and supporting tissue recovery). Despite its potential importance and role, very few studies specifically investigated CGRP in seizure and epilepsy models.

One preclinical study demonstrated that CGRP exerts neuroprotective effects through inhibition of the JAK1‐STAT1‐P2RX7 signaling axis.^8^ In detail, using a kainic acid‐induced epilepsy model generated by stereotaxic injection into the right hippocampus (1 μL at 250 ng/μL on three separate days) in C57BL/6 mice and glutamate induced neuronal injury models in HT22 and PC12 cells, the authors show that exogenous CGRP (administered by intraperitoneal injection after seizure induction at a dose of 50 μg/kg) significantly reduces seizure frequency and Racine scores, attenuates hippocampal neuronal loss, and decreases pro‐inflammatory cytokines including IL‐1β, IL‐6, and TNF‐α. Behavioral testing, including open field, novel object recognition, and Morris water maze, demonstrated that CGRP also improves cognitive impairment.

Mechanistically, seizures increased hippocampal P2RX7 expression at both mRNA and protein levels, whereas CGRP suppressed P2RX7 up‐regulation in vivo and in vitro. Pharmacological activation of P2RX7 reversed the protective effects of CGRP, while P2RX7 antagonism independently reduced seizure severity and neuronal damage.^8^

Upstream, the study identifies JAK1–STAT1 signaling as the regulatory pathway controlling P2RX7 expression; SE increased phosphorylation of JAK1 and STAT1, and CGRP inhibited this activation during the acute post‐SE phase. A STAT1 transcription enhancer reversed CGRP‐mediated P2RX7 suppression and neuroprotection, whereas a STAT1 inhibitor reduced P2RX7 expression and seizure severity. These data support a model in which CGRP suppresses neuronal P2RX7 expression by inhibiting JAK1‐STAT1 signaling, thereby reducing inflammation and neuronal death.^8^

Thus, CGRP may hypothetically have a dual role in epilepsy‐related pathology. Acutely, excess CGRP can promote neuronal firing and inflammation (potentially worsening seizures), but CGRP release also appears to be an adaptive response aiming to protect and repair neurons after seizure‐induced stress, showing a time‐dependent effect, precipitating hyperexcitable bursts and inflammation acutely, yet also engaging homeostatic mechanisms chronically. Furthermore, preclinical data also suggest a context‐dependent (peripheral vs. central) role of CGRP, with a peripheral protective effect when administered systemically in the kainic acid model, where it reduces seizure severity and neuronal damage. In contrast, increased central CGRP levels in key brain regions during kainic acid‐induced SE, following intrahippocampal or intracerebroventricular administration, appear to reflect a distinct central modulatory role.

## 5 THERAPEUTIC POTENTIAL OF ANTI‐CGRP TREATMENTS IN EPILEPSY

Two classes of anti‐CGRP drugs have been developed: anti‐CGRP or its receptor mAbs (erenumab, galcanezumab, fremanezumab, and eptinezumab) and gepants (rimegepant, ubrogepant, atogepant, and zavegepant).^1^ These agents efficiently inhibit CGRP signaling, resulting in decreased migraine frequency, but their role in epilepsy pathophysiology and seizure outcomes is unknown.

Preclinical results show that a CGRP receptor antagonist can prevent CGRP‐induced hyperexcitability. In the experiments performed on rat cortices mentioned earlier, the antagonist olcegepant (BIBN4096BS) abolished the epileptiform discharges and BBB disruption caused by CGRP application.^7^ This indicates that CGRP activity is indeed driving those epileptic bursts and inflammatory changes, and that blocking the CGRP receptor normalizes cortical activity. While this was an externally induced scenario (CGRP applied to cortex), it provides proof‐of‐concept that CGRP antagonism can counteract a pro‐seizure state directly in brain tissue. There are no animal models explicitly testing CGRP blockade in spontaneous epilepsy (e.g., whether giving a CGRP antibody after SE alters the development of chronic seizures). Interestingly, one study in fully kindled rats found that salmon calcitonin (a peptide precursor and related to CGRP) had anticonvulsant effects and even prevented epileptogenesis in the kindling model.^50^ While salmon calcitonin acts on calcitonin receptors (not CGRP receptors) and the mechanism may differ, this finding underlines the idea that modulating the calcitonin/CGRP peptide family may have a role in epileptogenesis in animal models.

Individuals who suffer from both epilepsy and migraine present a therapeutic challenge, as each condition can aggravate the other.^10^ Indeed, many ASMs also reduce migraine frequency. Notably, valproate and topiramate are FDA‐approved for migraine prophylaxis,^22^ exemplifying the overlap in mechanisms.

However, the possible effect of anti‐CGRP migraine therapies on seizure outcomes in individuals with comorbid migraine is unknown. Preliminary results from a large retrospective cohort study (using real‐world health records) compared PwE who were on CGRP inhibitors (presumably for migraine) against matched PwE not on anti‐CGRP drugs.^15^ The results showed that the group receiving anti‐CGRP therapy had a significant reduction in occurrence and likelihood of SE. Only 3.1% of PwE on CGRP inhibitors had an SE episode, versus 5.5% of those not on CGRP blockers, over the study period. This reflects roughly a halving of risk (risk ratio ~0.56) for severe, uncontrolled seizures in the anti‐CGRP group. Furthermore, hospital visits for SE were significantly less frequent in PwE on CGRP inhibitors than in the control group.^15^ The results should be interpreted keeping in mind several limitations, such as the lack of a fully peer‐reviewed publication detailing complete methodology, the retrospective use of an administrative database, potential misclassification bias from reliance on ICD‐coded SE outcomes, risk of confounding by indication due to systematic differences between treated and untreated groups, and inability to adjust for ASMs changes or other relevant covariates that may influence seizure risk. However, this is the first evidence that CGRP‐targeted treatments might confer an anti‐seizure benefit, particularly in preventing extreme seizure exacerbations like SE. The authors concluded that CGRP inhibitors should be considered as a treatment option in individuals who have both epilepsy and migraine, and they called for prospective trials to validate this therapeutic strategy.^15^

Beyond direct seizure control, anti‐CGRP agents might improve related aspects of epilepsy. For example, blocking CGRP could potentially alleviate certain types of headache that PwE experience (i.e., postictal headache), although it has not been investigated.^1^, ^51^

Moreover, certain newer ASMs like perampanel may themselves exhibit anti‐migraine effects via the CGRP pathway. Perampanel (an AMPA receptor non‐competitive antagonist) has been shown in vitro to inhibit both basal and KCl stimulated CGRP release from rat brainstem trigeminal circuits,^48^ which likely explains observed migraine relief in PwE on perampanel.^52^ However, a phase 2 RCT with a placebo group was conducted in 206 individuals to assess the efficacy and safety of perampanel in migraine prophylaxis (NCT00154063).^53^ During the titration phase, perampanel was initiated at 1.0 mg/day for 2 weeks, increased to 1.5 mg/day for the subsequent 2 weeks, and then further increased to 2.0 mg/day for 10 weeks. The last 2 weeks of titration continued into the 8‐week maintenance phase. The study was completed in 2015, but the results have not been published yet. However, results posted on Clinicaltrial.gov showed that from baseline to Week 19, both placebo and perampanel groups demonstrated a reduction in the mean number of migraine periods (a migraine headache that started, ended, or recurred within 24 or 48 h) per 28 days, irrespective of whether the 24‐ or 48‐h rule was used to define a migraine period.

Using the 24‐h rule, the mean change was −2.94 (SE 0.32; *n* = 104) in the placebo group and −2.73 (SE 0.39; *n* = 101) in the perampanel group. Using the 48‐h rule, the mean change was −3.31 (SE 0.39; *n* = 104) for placebo and −3.00 (SE 0.50; *n* = 101) for perampanel.^53^ The magnitude of improvement was comparable across groups, with placebo showing numerically slightly greater reductions under both definitions and overlapping standard errors suggesting no clear treatment separation; therefore, perampanel did not demonstrate superiority over placebo.

Overall, compared with modern migraine preventive trials, this study design more closely resembles earlier ASMs development paradigms than contemporary migraine‐specific methodology, also according to timing and endpoints recommended by the International Headache Society,^54^ with a design that might have limited the results.

Interestingly, a recent small open‐label study evaluated the effect of perampanel over 12 months on migraine attacks in 28 PwE and comorbid episodic migraine. Perampanel showed good effectiveness in reducing both epileptic seizures and migraine attacks, as well as analgesic intake at 6 months. These beneficial effects persisted and remained stable up to the 12‐month follow‐up in PwE and migraine, using a mean perampanel dose at 6‐ and 12‐month follow‐up of 5.5 ± 1.6 and 5.9 ± 1.9 mg/day, respectively.^52^

## 6 MONOCLONAL ANTIBODIES IN EPILEPSY AND THE ROLE OF BBB

Monoclonal antibodies (mAbs) represent a growing class of drugs with potential relevance to epilepsy through their ability to modulate inflammatory mediators implicated in seizure generation and epileptogenesis. However, their pharmacological limitation lies in restricted penetration across the intact BBB (as in migraine^55^). Under normal physiological conditions, less than 0.1% of circulating mAbs reach the CNS, and eventual successful delivery into the brain also does not preclude exit back to the systemic circulation.^51^, ^56^ This constraint raises questions about how such therapies could achieve neurological effects in epilepsy and CNS disorders with BBB integrity.^57^ Several efforts within a distinct and rapidly expanding field of research are specifically focused on developing and evaluating methods to enhance brain uptake of therapeutic antibodies.^57^, ^58^, ^59^

In inflammatory epilepsies, including autoimmune encephalitis, Rasmussen encephalitis, and new‐onset refractory SE (NORSE [new‐onset refractory status epilepticus] and FIRES [febrile infection‐related epilepsy syndrome]), BBB integrity is often compromised.^60^ Disruption of endothelial tight junctions and cytokine‐driven permeability allows larger molecules such as IgG‐based mAbs to enter the parenchyma and act directly on target immune cells or cytokine receptors within the CNS. For example, anti‐CD20 therapy with rituximab can deplete B cells trafficking across the permeable BBB in autoimmune epilepsies, correlating with reduced seizure burden.^61^ Small series report initial improvement in all treated individuals with seizure freedom sustained at 1–2 years in a subset, and additional single‐center experiences support the benefit, though evidence is observational and uncontrolled.^62^, ^63^

Similarly, IL‐6 receptor blockade with tocilizumab likely benefits NORSE and FIRES by suppressing intrathecal cytokine signaling during phases of barrier disruption. A NORSE series including seven PwE reported clinical improvement temporally associated with IL‐6 blockade with tocilizumab (4 mg/kg administered for 2 cycles in 1‐week intervals, with a monthly dose [8 mg/kg] added if needed),^64^ and a retrospective FIRES cohort (with one individual treated with tocilizumab, 8 mg/kg every 4 weeks) suggests reduced seizure frequency and intensity in this patient during the chronic phase.^65^

Anti‐TNF agents such as adalimumab may operate both peripherally, reducing systemic cytokine load, and centrally when BBB permeability is increased, leading to clinical seizure reduction in Rasmussen encephalitis administered 24 mg/m^2^ with a maximum of 40 mg via subcutaneous injection every 14 days, from 360 to 32 per quarter, with 45% meeting ≥50% response in an open pilot study, and follow‐up reports and a systematic review corroborate signal and tolerability.^66^

Finally, evidence for complement inhibition in epilepsy remains limited but indicative. Eculizumab, a C5 complement inhibitor, has demonstrated neurological improvement in complement‐mediated disorders and has been used off‐label in individuals whose epilepsy is secondary to such conditions.^67^, ^68^ However, epilepsy‐specific data are confined to isolated case reports without controlled studies. Overall, mAb‐based immunotherapies appear to reduce seizure activity when neuroinflammation or autoimmunity underlie network hyperexcitability, yet the current evidence derives mainly from small observational cohorts and case reports, with efficacy likely dependent on disease mechanism and timing of intervention.

Thus, while mAbs generally lack BBB penetration under normal conditions, the pathological breach of the barrier in inflammatory epilepsies provides a pharmacological window for efficacy.^69^ Their observed benefit in these contexts supports the concept that BBB dysfunction not only contributes to epileptogenesis but also temporarily permits therapeutic access for large‐molecule biologics targeting neuroimmune pathways.^70^

Finally, it should be noted that gepants, being small molecules, may penetrate more into the CNS, also with intact BBB, compared with anti‐CGRP mAbs, but still in a limited amount considering their pharmacokinetic properties.^1^, ^71^ However, it does not exclude the possibility of limited central effects in regions with reduced barrier integrity, such as the trigeminal nucleus caudalis, area postrema, and other circumventricular organs, nor does it rule out that very low central concentrations may exert minor pharmacodynamic effects. No data are currently available on gepants in PwE.

## 7 SEIZURE‐RELATED SAFETY PROFILES OF ANTI‐CGRP DRUGS

The safety profile of anti‐CGRP agents is generally favorable, as evidenced by migraine trials,^72^ observational studies,^73^ and post‐marketing surveillance.^74^, ^75^ These drugs do not cause systemic sedation, cognitive slowing, or organ toxicity that many ASMs do, which makes them attractive for adjunct therapy in epilepsy. In long‐term migraine studies, the most common adverse effects of CGRP mAbs have been injection site reactions, mild infections, and constipation (the latter especially with erenumab, and overall anti‐CGRP receptor antagonists).^1^ No significant increase in serious adverse events (such as hepatic or renal dysfunction) was observed compared with placebo in clinical trials.^1^ Importantly, no pro‐convulsant effects have been reported, thus blocking CGRP has not been observed to trigger seizures in individuals with migraine (and also in PwE and migraine as comorbidities) in clinical trials and observational studies.^72^, ^73^

In regulatory documents from the FDA, including the clinical safety review for fremanezumab, a single case of a generalized seizure was reported during the clinical development program. In this case, EEG showed generalized epileptiform discharges, brain MRI was unremarkable, levetiracetam was initiated, and nortriptyline (as another preventive treatment for migraine) was discontinued. The investigator considered the seizure possibly related to fremanezumab in the context of a previously unrecognized epileptic disorder.^76^

Post‐marketing pharmacovigilance data provides complementary insights into safety, bearing in mind their intrinsic limitations (*see below*). Regarding anti‐CGRP mAbs, in the most recent FAERS (FDA Adverse Event Reporting System) disproportionality study^77^ covering years from 2018 to 2023 (65 792 total reports: erenumab 38 515; galcanezumab 19 485; fremanezumab 5332; eptinezumab 2460),^77^ no cases of seizures have been reported.^77^

The last post‐marketing pharmacovigilance data on gepants^75^ included more than 10 000 reports, which assessed both VigiAccess and FAERS through March 2024 (zavegepant had minimal VigiAccess entries and no FAERS mining at that cutoff). In the disproportionality analysis, seizure emerged as a detectable safety signal for both atogepant and ubrogepant, although absolute case numbers were low and the magnitude of association was modest. In fact, seizure was reported in 40 cases (1.09%) for atogepant and 18 cases (0.92%) for ubrogepant. For atogepant, the signal met standard criteria (ROR 1.76; ROR_0–25_ 1.29). For ubrogepant, the association was weaker and borderline (ROR 1.64; ROR_0–25_ 1.03).^75^

However, several limitations constrain interpretation. First, FAERS is a spontaneous reporting system and lacks denominator data, preventing estimation of incidence rates. Second, disproportionality reflects reporting imbalance rather than causality. Third, case numbers were small, increasing susceptibility to statistical instability. Fourth, confounding by indication is possible, as migraine populations may have underlying epilepsy as a comorbidity. Fifth, clinical details such as seizure type, prior epilepsy history, imaging findings, or medication adjustments are unavailable. Therefore, while seizure appears as a detectable signal for atogepant and ubrogepant in pharmacovigilance analyses, the low absolute counts and intrinsic limitations of spontaneous reporting databases preclude causal inference. Further evaluation using controlled observational datasets or prospective surveillance would be required to clarify any clinical relevance.

Importantly, seizure is not listed in the FDA and/or EMA labeling for rimegepant, atogepant, ubrogepant, or zavegepant, suggesting that it is not a recognized regulatory safety signal at present.

## 8 POSSIBLE ROLE OF CGRP ACROSS EPILEPSY SUBTYPES AND SPECIAL POPULATIONS

One relevant question is whether CGRP and anti‐CGRP therapies affect different epilepsy subtypes in distinct ways. Epilepsy is heterogeneous and includes generalized and focal forms, and it is not known if CGRP's role is more relevant in one versus the other.

To date, most direct evidence for CGRP involvement comes from preclinical focal epilepsy models, especially those with significant tissue injury or inflammation. The kainic acid model (a limbic SE leading to temporal lobe epilepsy) showed pronounced CGRP upregulation, as noted above.^4^ Similarly, cortical application of CGRP causing local epileptiform activity is essentially a focal cortical excitation phenomenon.^6^, ^7^ These findings suggest that in focal epileptogenesis, such as after a brain insult, in traumatic or mesial temporal lobe epilepsy, CGRP may be a mediator of the subsequent hyperexcitable, inflamed state. In contrast, primary generalized epilepsies (like genetic generalized epilepsy) may not engage the CGRP pathway as strongly.

For instance, the absence of CGRP increase after a single generalized tonic–clonic seizure in the PTZ model suggests that brief global seizures (without focal injury) do not elicit the same CGRP‐driven inflammatory response. Thus, people with generalized epilepsy might see less direct impact from modulating CGRP. That said, generalized epilepsies can still involve systemic stress (e.g., hypoxia, widespread neuronal firing), which could trigger CGRP release. No study to date has stratified the effect of anti‐CGRP treatments by epilepsy subtype. Importantly, the large observational study on SE included a mix of PwE and found a benefit of CGRP inhibitors overall, implying that even if CGRP is more important in focal cases, the aggregate effect in a mixed population was still positive.

The presence of migraine in a PwE likely marks a phenotype where CGRP‐related mechanisms are upregulated. These individuals often have a more prominent trigeminovascular activation component; for example, they may experience preictal or postictal headache or migraine‐triggered seizures. Thus, CGRP‐targeted therapy may directly address migraine and potentially interrupt a migraine‐seizure facilitation cycle; this remains hypothetical.

Conversely, the role of anti‐CGRP therapies in PwE without comorbid migraine remains unclear. It is conceivable that a subset of PwE, even in the absence of overt migraine, may exhibit elevated CGRP levels, but the clinical implications of this observation require further investigation. It is worth noting that migraine and epilepsy share some genetic and molecular pathways (ion channels, neurotransmitter systems, neuroinflammatory mediators), reinforcing that a therapy impacting one may influence the other.

## 9 LIMITATIONS AND CHALLENGES

There have been no RCTs of anti‐CGRP drugs in PwE to date, and available preclinical evidence supporting a genuine antiepileptogenic effect remains limited, as it derives almost exclusively from acute, short‐term preclinical models lacking chronic epileptogenesis endpoints and limited testing with the anti‐CGRP agents currently used in clinical practice.^6^ It therefore remains to be seen whether CGRP blockade would significantly impact baseline seizure frequency in a controlled clinical setting, and dose optimization and choice of drug (mAb vs. gepant) for epilepsy remain unexplored. Theoretically, CGRP blockade may attenuate seizure‐related hyperexcitability and neuroinflammation, but it could also interfere with endogenous CGRP‐mediated repair and recovery mechanisms after seizures; moreover, its effects across different epilepsy subgroups remain unknown. A further, largely unresolved issue concerns CNS penetration: mAbs act primarily outside the brain, so it is unclear whether a peripherally acting CGRP blocker sufficiently affects central seizure‐related mechanisms, with some effects possibly mediated via trigeminal‐cortical reflexes and vascular routes; gepants, as small molecules, may achieve somewhat greater CNS penetration, though still in a limited amount. Taken together, the mechanistic hypotheses discussed in this review should be regarded as hypothesis‐generating and require confirmation in dedicated preclinical studies with chronic designs before any translational inference can be drawn.

## 10 CONCLUSION

Preclinical evidence shows that CGRP may play a dual role, promoting cortical hyperexcitability and inflammation while also activating neuroprotective and reparative mechanisms. Experimental evidence, with limited preliminary clinical support, suggests that excessive CGRP signaling may contribute to seizure generation, particularly in models involving neuroinflammation or BBB disruption. It is important to emphasize that the mechanistic hypotheses discussed above are largely derived from acute or short‐term preclinical models, and that robust evidence supporting a disease‐modifying or antiepileptogenic effect of CGRP‐targeting therapies is currently lacking.

## AUTHOR CONTRIBUTIONS

MR and LFI supervised the review, performed the screening for studies, and wrote the initial manuscript. All Authors critically reviewed the manuscript, agreed to be fully accountable for ensuring the integrity and accuracy of the work, and read and approved the final manuscript.

## FUNDING INFORMATION

This research did not receive any specific grants from funding agencies in the public, commercial, or not‐for‐profit sectors.

## CONFLICT OF INTEREST STATEMENT

MR has received research support and support to attend national and international conferences from Angelini Pharma, Lundbeck, Jazz Pharmaceuticals, and AbbVie. Currently conducting the “Epilepsia Open internship.” LFI received financial support, consulting fees for the participation in advisory boards, and support for attending meetings from: Teva, Eli Lilly, Lundbeck, Pfizer, Organon, and AbbVie; he is Associate Editor for *Frontiers in Neurology* and junior editor for *Confinia Cephalagica*, *Cephalalgia*, and *Cephalalgia Report*. LP is/has been a consultant or advisory board member for: AbbVie, USA; BCG, Switzerland; Boehringer Ingelheim International GmbH, Germany; GH‐Research, Ireland; Immunogen, USA; Johnson & Johnson USA; LB Pharmaceuticals, USA; Magdalena BioSciences, USA; Sanofi‐Aventis‐Genzyme, France and USA; Lundbeck, Denmark and Italy; Napo‐Pharma, USA and EU; NetraMark, Canada; Pfizer Global, USA; Relmada Therapeutics, USA; Takeda, USA and owns shares/options from AiDvance Germany; Adapt UK, Enzeta USA, NetraMark Canada, and Relmada, USA. CV has received research support, speaker honouraria and support to attend national and international conferences from Lundbeck, Pfizer and AbbVie, Jazz Pharmaceuticals and Angelini Pharma. PC has received research support, speaker honouraria and support to attend national and international conferences from AbbVie, Bayer Schering, Bial, Biogen‐Dompè, Biogen‐Idec, Eisai, Genzyme, Lundbeck, Lusofarmaco, Merck‐Serono, Novartis, Prexton, Teva, UCB Pharma and Zambon.

Other authors have no relevant financial or non‐financial interests to disclose.

## ETHICS STATEMENT

We confirm that we have read the Journal's position on issues involved in ethical publication and affirm that this report is consistent with those guidelines.

## Data Availability

No new data were generated.
